# Frovatriptan plus dexketoprofen in the treatment of menstrually related migraine: an open study

**DOI:** 10.1007/s10072-013-1390-0

**Published:** 2013-05-22

**Authors:** Gianni Allais, Sara Rolando, Paola Schiapparelli, Gisella Airola, Paola Borgogno, Ornella Mana, Chiara Benedetto

**Affiliations:** Women’s Headache Center, Department of Gynecology and Obstetrics, University of Turin, Via Ventimiglia 3, 10126 Turin, Italy

**Keywords:** Dexketoprofen, Frovatriptan, Menstrually related migraine

## Abstract

At least 50 % of female migraineurs experience migraine associated with the perimenstrual period, even though they may also suffer from attacks at other times of the cycle (menstrually related migraine, MRM). MRM attacks tend to be longer and more intense than those arising in other phases of the menstrual cycle, and are often aggravated by more pronounced vegetative phenomena. In this open preliminary trial, we tested the efficacy of associating frovatriptan and dexketoprofen for the treatment of an acute attack of MRM, diagnosed according to the criteria of the International Headache Society, in 24 patients between 19 and 45 years of age (mean 31.33 ± 7.33). Twenty-one of them completed the study. Pain relief was achieved by 76 % of patients at 2 h and by 86 % at 4 h. A pain-free state was achieved by 48 % at 2 h and by 62 % at 4 h from taking the product. A pain-free state at 24 h was present in 76 % of MRM sufferers, 33 % of whom showed a sustained pain-free state at 24 h. A rescue medication was needed by eight patients. While decidedly encouraging, the data of this study obviously need confirmation with double blind studies involving a greater number of patients.

## Introduction

Migraine predominantly affects the female sex, with a female to male ratio of approximately 3:1 [1, 2]. One of the important reasons for this unbalanced distribution between the sexes is that the menstrual cycle, with its characteristic fluctuations in sex hormones, is among the main factors triggering migraine attacks [3].

Menstrual migraine (MM) is a highly prevalent condition associated with considerable disability. At least 50 % of female migraineurs experience migraine associated with the perimenstrual period, even though they may also suffer from attacks at other times of the cycle (menstrually related migraine, MRM) [4]. Most of the data in the literature report that attacks of MRM tend to be longer and more intense than those arising in other phases of the menstrual cycle, and are often aggravated by more pronounced vegetative phenomena (e.g., nausea, vomiting, and photo-phonophobia) [5]. As a result, MRM attacks are much more disabling than non-menstrual ones and can be particularly difficult to treat.

Triptans are recommended as the first-line treatment for moderate to severe migraine attacks [6], including MRM [7]. It should be remembered, however, that prostaglandins (PGs) play a very important role in the genesis of pain associated with menstruation, including migraine [8]. Therefore, the hypothesis that much greater benefit can be achieved in treating MM attacks by combining a non-steroidal anti-inflammatory drug (NSAID) with a triptan is very interesting.

This was tested by combining 85 mg sumatriptan and 500 mg naproxen sodium in a single tablet, which was proposed as a way to potentiate the therapeutic effect in cases of MRM [9]. The efficacy and tolerability of this combination drug was evaluated in two RCTs for a total of 621 women suffering from MRM in association with dysmenorrhea. The rate of sustained pain-free response at 2 and 24 h was statistically higher for sumatriptan–naproxen compared with placebo. Moreover, this drug combination also relieved non-painful menstrual symptoms.

Frovatriptan, one of the newest triptans, has a high affinity for 5-HT_1B/1D_ receptors and a long half-life, both of which contribute to its distinctive clinical effect of having a more sustained and prolonged action than other triptans. Indeed, frovatriptan not only proved effective for treating the acute attack of MM, but particularly for short-term preventive therapy of MM [10]. In addition, frovatriptan is one of the safest triptans, with the lowest risk of causing adverse events. Following extensive evidence from randomized pharmacological trials, frovatriptan has now earned a grade A recommendation from guidelines for short-term prophylaxis of MM [11]. Recent post hoc analyses of direct comparative trials also suggested that frovatriptan might play an important role in the acute treatment of MRM. In these studies, frovatriptan showed pain relief and pain-free rates similar to those of zolmitriptan, rizatriptan, and almotriptan, but with significantly lower recurrence rates [12].

Dexketoprofen is a NSAID that blocks the action of cyclo-oxygenase, which is involved in PGs production, thus reducing inflammation and pain [13]. The *t* max and half-life of dexketoprofen are 30 min and 2 h, respectively. We have previously demonstrated in 42 women affected by migraine with or without aura that dexketoprofen very quickly reduces pain intensity and accompanying symptoms [14].

Based on the pharmacological properties of the two products, both of which have already proven to be effective in treating migraine, we decided to test the combination of frovatriptan and dexketoprofen, in an open preliminary trial, to determine the efficacy of using them together for the treatment of an acute attack of MRM.

## Patients and methods

The study involved 24 patients between 19 and 45 years of age (mean age 31.33 ± 7.33; mean age at onset of migraine 19.37 ± 9.81) from the Women’s Headache Center of the University of Turin, and suffering from migraine associated with menstruation (menstrually related migraine, MRM), diagnosed according to the criteria of the International Headache Society (ICHD-II, 2004). The patients were told to treat an attack of MRM by self-administering simultaneously a tablet of 2.5 mg frovatriptan and one tablet of 25 mg dexketoprofen, as soon as possible from the beginning of the migraine attack. If the frovatriptan + dexketoprofen combination proved to be ineffective, the patients were allowed to assume a rescue medication for pain, provided that it be taken at least 2 h later. The patients filled out a diary that was specially designed to highlight the characteristics of the migraine pain during the course of treatment.

Reported here are the results regarding the pain relief state and the pain-free condition at 2 and 4 h from commencing treatment. In addition, data about the pain-free state at 24 h and the sustained pain-free state at 24 h are pointed out.

## Results

**Fig. 1:**
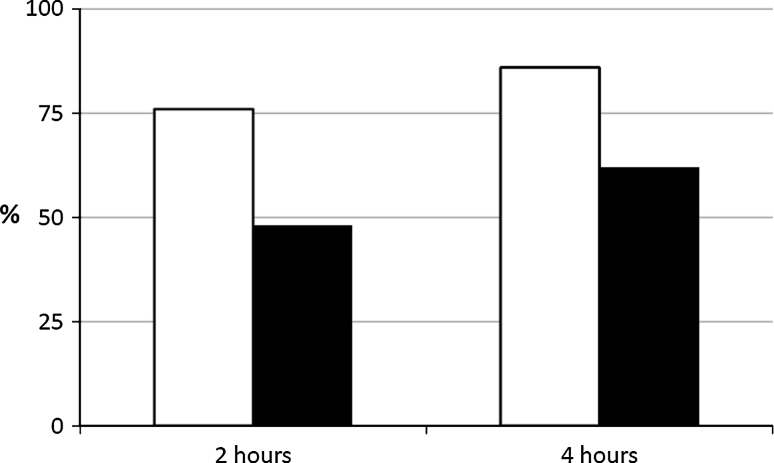
Percentage of patients who achieved pain relief (*white columns*) and were pain free (*black columns*) after frovatriptan plus dexketoprofen treatment at 2 and 4 h

Two patients filled in the treatment diary incorrectly and one patient failed to come to the post-treatment follow-up and were, thus, excluded from the data analysis, which therefore took into account 21 patients. Pain relief was achieved by 16 women (76 %) at 2 h and by 18 women (86 %) at 4 h (Fig. 1).

A pain-free state was achieved by ten patients (48 %) at 2 h and by 13 patients (62 %) at 4 h from taking the product (Fig. 1). A pain-free state at 24 h was present in 16 women (76 %), seven of whom (33 %) showed a sustained pain-free state at 24 h. A rescue medication was needed by eight patients (38 %). None of the patients reported serious side effects due to taking frovatriptan and dexketoprofen together.

## Discussion

Considering that MRM attacks are particularly difficult to treat, the results achieved by associating frovatriptan 2.5 mg with 25 mg dexketoprofen appear very encouraging. What is particularly significant is the number of women who were already reporting a pain-free state at 4 h (64 %) and even more remarkably at 24 h (76 %). Comparative studies of other triptans were used for treating, an acute attack of MRM showed that frovatriptan is highly effective when used alone it produced a pain-free state in 52 % of the patients at 4 h, and in 66 % of the patients at 24 h [15]. Therefore, if the above result is compared with the percentages of frovatriptan’s effectiveness obtained in the present trial, it becomes quite clear that added benefit can be derived from combining dexketoprofen with frovatriptan rather than using frovatriptan alone. While decidedly encouraging, the data of this study obviously need confirmation with double-blind studies involving a greater number of patients.

## Abbreviations

MM: Menstrual migraine
MRM: Menstrually related migraine
PGs: Prostaglandins
